# Spinning carbon and sinking phosphorus: Misaligned cycles in the sea

**DOI:** 10.1073/pnas.2602952123

**Published:** 2026-03-23

**Authors:** Matthew J. Church, Katie N. Coates

**Affiliations:** ^a^Flathead Lake Biological Station, University of Montana, Polson, MT 59860

Each year, the ocean absorbs more than 2 Pg of anthropogenic CO_2_, roughly a third of human emissions (1), but the climatic value of this uptake depends on how long that carbon remains isolated from the atmosphere. Proposals to fertilize large ocean regions aim to enhance photosynthesis and transfer newly formed organic matter into the interior sea. A central metric for judging the effectiveness of such strategies is therefore the duration of storage (2). Yet, export is sustained, and ultimately limited, by essential nutrients that are recycled by marine organisms and later returned by circulation. How quickly these nutrients are returned sets the pace at which additional carbon can be captured again. Sullivan et al. (3) use an inverse biogeochemical ocean circulation model to examine time scales of carbon and phosphorus cycling, finding that carbon is remineralized and returned to the surface ocean faster than the essential nutrient phosphorus. Misalignment in timing of these cycles has important potential consequences for fertilization-based marine CO_2_ removal proposals.

The ocean functions as a massive bioreactor where diverse microorganisms catalyze the flow of energy and the cycling of matter. In the sunlit upper ocean, photosynthetic microbes assimilate CO_2_ together with inorganic nutrients (e.g., nitrogen, phosphorus), producing ~55 Pg C yr^−1^ of organic matter, nearly half of global net primary production. Most of the recently produced organic matter is consumed through upper ocean food webs, leaving a relatively small fraction (5 to 20%) available for export into the interior ocean. Export can occur through various mechanisms, including sinking particles, vertically migrating animals, and transport by physical circulation (4). Once in the interior waters, exported organic matter fuels the metabolism of diverse consumers, recycling CO_2_ and nutrients. These recycled byproducts are eventually returned to the upper ocean by circulation.

Time scales of ocean carbon storage depend critically on the form of organic matter produced. While material packaged into particles can settle rapidly to depth via gravitational sinking, dissolved carbon is transported by circulation. A large fraction (50% or more) of newly produced primary production flows through highly reactive dissolved organic matter pool, supporting the metabolism of abundant and diverse heterotrophic bacteria (5). Although only a modest fraction of primary production is exported via sinking particles, particles transport this carbon into reservoirs that are isolated from atmospheric exchange for decades or longer. The larger share of primary production routed into dissolved organic matter gets remineralized at depths where waters reconnect with the surface on far shorter timescales (<1 y). Hence, biology is doing more than just moving carbon out of atmosphere and into the interior ocean: It is also sorting organic matter into different pools, each with distinct lifetimes.

Within the dissolved organic matter pool, there is a continuum of compounds with different susceptibility to remineralization. To simplify this geochemical diversity, dissolved organic matter is often binned into operational classes based on its reactivity and residence times. Labile compounds cycle within hours to days, semilabile material can persist for months to years, and the diagenetically altered refractory constituents resist microbial degradation for centuries or longer. Importantly, the longer-lived refractory pool accumulates organic matter very slowly, after progressive biological modification of organic matter as it moves through the remineralization intensive particulate, labile, and semilabile pools. This hierarchy of persistence further reinforces the message of Sullivan et al. (3): Only organic matter that escapes the fast, carbon-rich pathways contributes meaningfully to long-term sequestration.

Because dissolved and particulate organic matter differ in composition, the partitioning of production between these pools has inevitable stoichiometric consequences important to long-term storage. Compared with particles, dissolved organic matter in the upper ocean is enriched in carbon relative to phosphorus, reflecting both its production and the preferential removal of nutrients during microbial processing (6, 7). When this carbon-rich material is remineralized within waters that exchange with the surface ocean relatively quickly, the resulting CO_2_ can exchange with the atmospheric sooner than nutrients recycled during remineralization (Fig. 1). In contrast, particulate material can be more enriched in phosphorus (lower carbon-to-phosphorus ratios) and downward sinking particles store these nutrients over longer time scales. Increasing residence time yields a storage inventory that becomes progressively enriched in phosphorus relative to carbon. The longer carbon and nutrients remain isolated, the further their proportions diverge from those of surface production, biasing long-term storage toward nutrient-rich, carbon-poor byproducts.

**Fig. 1.**
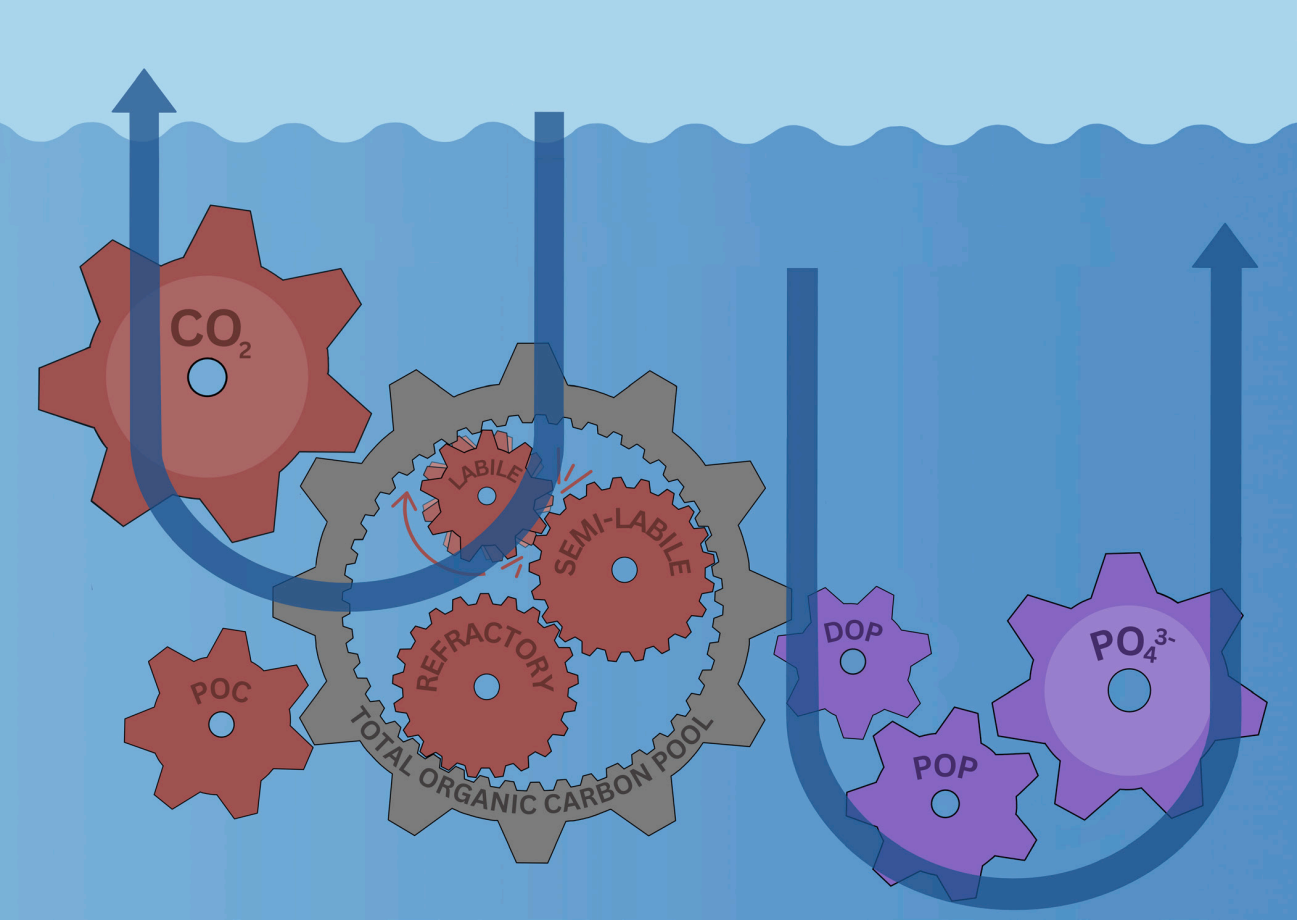
The flow of organic matter into different forms determines carbon and phosphorus storage times in the ocean. Surface production is partitioned between carbon-rich dissolved organic matter and particulate organic matter. Organic carbon that flows into labile dissolved organic carbon is rapidly remineralized in waters with faster reconnection to the surface ocean, while phosphorus is largely partitioned into particulate organic matter that can settle in waters with slower recirculation to the surface. Abbreviations are POC = particulate organic carbon; POP = particulate organic phosphorus; DOP = dissolved organic phosphorus.

The proposed stoichiometric drift becomes particularly consequential when considering ocean fertilization as a tool to increase biological carbon uptake. Enhancing production, whether through natural variability or deliberate intervention, moves organic matter through pathways that can recycle carbon and nutrients on different timelines. This could lead to what Sullivan et al. term a “productivity hangover,” where remineralized CO_2_ rapidly revisits the surface, while phosphorus remains trapped in longer circulation trajectories. Short-term delivery of nutrient-deficient waters would suppress further carbon uptake, posing challenges to fertilization-based marine carbon dioxide removal strategies (8, 9). However, on longer time scales, eventual circulation of phosphorus-enriched waters to the surface ocean could reorganize regional patterns of nutrient limitation and microbial community structure. Because organic carbon and nitrogen tend to be more closely coupled than phosphorus, it is likely that faster remineralization of organic carbon might also apply to organic nitrogen, adding further complexity to downstream biogeochemical consequences of the misalignment in elemental cycles. Excess supply of phosphorus relative to nitrogen would likely favor N_2_ fixing microorganisms whose activities would restore nitrogen but consume phosphorus (10). The slower return of phosphorus compared to carbon or nitrogen could alter regional nutrient inventories and partially reset the conditions that gave rise to the original imbalance. In this way, the time-integrated carbon export would depend on both the magnitude of initial export and also the history of decoupled elemental cycling experienced by the waters as they move through the interior ocean.

The analysis of Sullivan et al. should rekindle long-standing interests in improving understanding of processes structuring the stoichiometry of remineralization (11, 12). Observational and modeling studies have alternately found that organic phosphorus or carbon is recycled more rapidly, often based on depth-dependent changes in the stoichiometry of particles and dissolved organic matter (13–15). Models highlight sensitivity in atmospheric CO_2_ to regional-scale variations in organic matter carbon-to-phosphorus ratios (16), for example, due to production of high carbon-to-phosphorus material in places where surface phosphate concentrations are low (16). However, if stoichiometric drift occurs as predicted by Sullivan et al. (3), this would dampen the impacts associated with export of high carbon-to-phosphorus ratio organic matter on carbon storage.

Time-dependent misalignment in carbon and nutrient cycles also offers a possible mechanism for negative preformed nutrient anomalies described throughout the subtropical gyres (17, 18). Waters in these regions are highly depleted in nutrients due to rapid biological uptake and low supply. But if labile sources of carbon are remineralized faster than phosphorus, as Sullivan et al. argue, this would consume oxygen more quickly than nutrients are replenished to these waters, resulting in anomalously nutrient-poor waters relative to oxygen deficits. From this perspective, the anomalous stoichiometric behavior of the low latitude gyre thermoclines could be viewed as the downstream signature of stoichiometric drift where carbon leaks back toward the atmosphere through faster loops, while phosphorus remains trapped in longer recycling loops.

Sullivan et al. (3) use an inverse biogeochemical ocean circulation model to examine time scales of carbon and phosphorus cycling, finding that carbon is remineralized and returned to the surface ocean faster than the essential nutrient phosphorus.

An important implication of Sullivan et al. is that evaluating ocean carbon storage requires attention to when elements return to the surface, not simply the depth of export. Because export is governed by processes operating across a hierarchy of timescales, from microbial transformations occurring over hours to days, to circulation pathways that operate on annual to centennial scales, understanding biological carbon storage demands robust coordination between observations and models. Notably, there is a clear need for improved understanding of processes that underpin remineralization and how these processes shape stoichiometry. For example, measurements of sinking particles and dissolved organic matter frequently show increasing carbon- to-phosphorus ratios with age, consistent with preferential removal of phosphorus (6, 7, 19, 20). Determining how such selective fractionation shapes long-term storage requires integration of time-resolved observations and models that follow elements along their circulation trajectories. In addition, better understanding the processes that sort organic matter into pools of differing reactivity would improve their representation in models. To be successful at long-term storage of carbon, fertilization approaches would need to circumvent processes that funnel carbon into more reactive dissolved pools and in favor of those that emphasize production of fast-sinking particles.

## References

[1] T. DeVries , Magnitude, trends, and variability of the global ocean carbon sink from 1985 to 2018. Glob. Biogeochem. Cycles **37**, e2023GB007780 (2023).

[2] K. O. Buesseler , The case for ocean iron fertilization field trials. Dialogues Clim. Change, 10.1177/29768659261420631 (2026).

[3] M. R. Sullivan , Decoupled timescales of organic carbon and phosphorus recycling in the global ocean. Proc. Natl. Acad. Sci. U.S.A. **123**, e2514991123 (2026). 41701833 10.1073/pnas.2514991123 PMC12933148

[4] P. W. Boyd, H. Claustre, M. Levy, D. A. Siegel, T. Weber, Multi-faceted particle pumps drive carbon sequestration in the ocean. Nature **568**, 327–335 (2019). 30996317 10.1038/s41586-019-1098-2

[5] M. A. Moran , The Ocean’s labile DOC supply chain. Limnol. Oceanogr. **67**, 1007–1021 (2022).

[6] C. S. Hopkinson, J. J. Vallino, Efficient export of carbon to the deep ocean through dissolved organic matter. Nature **433**, 142–145 (2005). 15650735 10.1038/nature03191

[7] A. N. Loh, J. E. Bauer, Distribution, partitioning and fluxes of dissolved and particulate organic C, N and P in the eastern North Pacific and Southern Oceans. Deep Sea Res. I Oceanogr. Res. Pap. **47**, 2287–2316 (2000).

[8] National Academies of Sciences, Engineering, and Medicine, A Research Strategy for Ocean-based Carbon Dioxide Removal and Sequestration (The National Academies Press, 2022). 35533244

[9] A. Oschlies, W. Koeve, W. Rickels, K. Rehdanz, Side effects and accounting aspects of hypothetical large-scale Southern Ocean iron fertilization. Biogeosciences **7**, 4017–4035 (2010).

[10] D. Karl, R. Letelier, Nitrogen fixation-enhanced carbon sequestration in low nitrate, low chlorophyll seascapes. Mar. Ecol. Prog. Ser. **364**, 257–268 (2008).

[11] E. J. Zakem, N. M. Levine, Systematic variation in marine dissolved organic matter stoichiometry and remineralization ratios as a function of lability. Glob. Biogeochem. Cycles **33**, 1389–1407 (2019).

[12] C. Deutsch, T. Weber, Nutrient ratios as a tracer and driver of ocean biogeochemistry. Annu. Rev. Mar. Sci. **4**, 113–141 (2012). 10.1146/annurev-marine-120709-142821 22457971

[13] S. D. Gerace , Depth variance of organic matter respiration stoichiometry in the subtropical North Atlantic and the implications for the global oxygen cycle. Glob. Biogeochem. Cycles **37**, e2023GB007814 (2023).

[14] R. T. Letscher, J. K. Moore, Preferential remineralization of dissolved organic phosphorus and non-Redfield DOM dynamics in the global ocean: Impacts on marine productivity, nitrogen fixation, and carbon export. Glob. Biogeochem. Cycles **29**, 325–340 (2015).

[15] J. H. Martin, G. A. Knauer, D. M. Karl, W. W. Broenkow, VERTEX: Carbon cycling in the northeast Pacific. Deep Sea Res. I Oceanogr. Res. Pap. **34**, 267–285 (1987).

[16] E. D. Galbraith, A. C. Martiny, A simple nutrient-dependence mechanism for predicting the stoichiometry of marine ecosystems. Proc. Natl. Acad. Sci. U.S.A. **112**, 8199–8204 (2015). 26056296 10.1073/pnas.1423917112 PMC4500256

[17] S. E. Fawcett, K. S. Johnson, S. C. Riser, N. Van Oostende, D. M. Sigman, Low-nutrient organic matter in the Sargasso Sea thermocline: A hypothesis for its role, identity, and carbon cycle implications. Mar. Chem. **207**, 108–123 (2018).

[18] K. S. Johnson, S. C. Riser, D. M. Karl, Nitrate supply from deep to near-surface waters of the North Pacific subtropical gyre. Nature **465**, 1062–1065 (2010). 20577212 10.1038/nature09170

[19] R. K. Foreman, K. M. Björkman, C. A. Carlson, K. Opalk, D. M. Karl, Improved ultraviolet photo-oxidation system yields estimates for deep-sea dissolved organic nitrogen and phosphorus. Limnol. Oceanogr. Methods **17**, 277–291 (2019).

[20] G. A. Knauer, J. H. Martin, K. W. Bruland, Fluxes of particulate carbon, nitrogen, and phosphorus in the upper water column of the Northeast Pacific. Deep Sea Res. I Oceanogr. Res. Pap. **26**, 97–108 (1979).

